# Evaluation of the association between haemoglobin levels and preterm birth at Khartoum, Sudan: A hospital-based study

**DOI:** 10.3389/fnut.2022.933557

**Published:** 2022-07-22

**Authors:** Abdelmageed Elmugabil, Nadiah M. Alhabrdi, Duria A. Rayis, Osama Al-Wutayd, Ishag Adam

**Affiliations:** ^1^Faculty of Medicine, El Imam El Mahdi University, Kosti, Sudan; ^2^Department of Obstetrics and Gynecology, Unaizah College of Medicine and Medical Sciences, Qassim University, Unaizah, Saudi Arabia; ^3^Faculty of Medicine, University of Khartoum, Khartoum, Sudan; ^4^Department of Family and Community Medicine, Unaizah College of Medicine and Medical Sciences, Qassim University, Unaizah, Saudi Arabia

**Keywords:** haemoglobin, preterm, birth, Sudan, anaemia

## Abstract

**Objective:**

The objective of this study was to determine the association between haemoglobin level and PB.

**Methods:**

A cross-sectional study was conducted in Khartoum, Sudan. Questionnaires on demographics and medical and obstetric factors were completed. A logistic regression analysis was performed.

**Results:**

Of the 1,716 pregnant women, approximately two-thirds (65.7%) had anaemia (haemoglobin < 11 g/dl) and six (0.3%) had severe anaemia (haemoglobin < 8 g/dl). Of the 1,716 women, 283 (16.5%) had a PB. In multivariable logistic regression, parity (AOR = 1.15, 95% CI = 1.09–1.21, *P* < 0.001) was positively associated with PB. Compared to those with haemoglobin levels of 10–10.9 g/dl, pregnant women with haemoglobin levels of 8–8.9 (AOR = 0.41, 95% CI = 0.22–0.77), 9–9.9 (AOR = 0.59, 95% CI = 0.38–0.91), and 11–11.9 g/dl (AOR = 0.53, 95% CI = 0.36–0.77) were at a lower risk of PB. Women with haemoglobin levels of 12–13 g/dl were at a higher risk of PB (AOR = 1.62, 95% CI = 1.06–2.45). There was no significant association between women with haemoglobin levels < 8 g/dl and > 13 g/dl and PB.

**Conclusion:**

This study showed different levels of association between haemoglobin levels and PB.

## Introduction

Preterm birth (PB) refers to the birth of a baby before 37 completed weeks of gestation or 259 days from the final day of the last menstrual period (1). PB is a worldwide health problem that affected 14.84 million births in 2014 (2). More than three-quarters (81.1%) of all PBs occur in Africa and South Asia (2). PB is a major cause of perinatal death and a significant cause of long-term consequences among the survivors (3). PB is the main direct cause of neonatal death and is associated with 50–75% of all neonatal mortality and half of all neonatal morbidity worldwide (4). PB also presents an economic burden, due to the requirement of neonatal intensive care units, and is associated with socioeconomic disadvantages and disruptive life events during pregnancy (5). Recent studies showed a high prevalence of PB in sub-Saharan African countries (6, 7). Several factors such as age, short interpregnancy interval (6, 8), being a rural resident, inadequate antenatal care (9), previous PB, multiple pregnancies, and malaria (6) are significantly associated with PB. It is of paramount importance that the factors associated with PB (especially haemoglobin) in different settings are well documented, if the goal of the WHO and United Nations 2010 of reducing mortality due to PB by 50% before 2025 is to be achieved. Risk factors for PB should be correctly identified and properly managed to reduce the incidence of PB. Maternal nutrition may impact both haemoglobin synthesis and foetal growth, development, survival, and PB (10, 11). Previous studies on the association between the haemoglobin level and PB showed inconsistent results. While some found that anaemia was associated with PB (8, 12), others showed that a high haemoglobin level carries an increased risk of PB (13, 14). Although the prevalence of PB is high in many African countries (6, 7), pertinent information on the association between haemoglobin level and PB and spontaneous PB is not adequately documented in sub-Saharan Africa, including Sudan. This study was conducted to investigate the association between the haemoglobin level and spontaneous PB in Khartoum, Sudan.

## Materials and methods

A cross-sectional study was conducted at Saad Abuelela Maternity Hospital in Khartoum, Sudan, from February to November 2020. The inclusion criteria were pregnant women with a single live baby. The exclusion criteria were post-term birth (≥ 42 weeks of gestation), unknown gestational age, women with unknown body mass index (BMI) in early pregnancy, seriously ill women, multiple births, stillbirths, and congenital malformed deliveries. After signing an informed consent form, trained medical residents conducted face-to-face interviews with the pregnant women included in the study. Questionnaires on demographics and medical and obstetric factors were filled out in the local language (Arabic). The questionnaires recorded information concerning the age, parity, education, residence, occupation, antenatal care status, history of previous miscarriages/PBs, gestational age, interpregnancy interval, haemoglobin level, and infant’s sex. Gestational age was calculated using a combination of the dates of the last menstrual period and early pregnancy ultrasound. PB refers to the birth of the baby before 37 completed weeks of gestation. Additional information was extracted from the clinical notes on pregnancy complications, such as data on hypertension, preeclampsia, or diabetes (defined as gestational or chronic). Early pregnancy (< 14 weeks) weight and height were used to calculate BMI as weight in kilograms divided by the squared height in metres. The WHO classification was used to group the women, according to their BMIs, like normal weight (18.5–24.9 kg/m^2^), overweight (25–29.9 kg/m^2^), or obese (30–34.9 kg/m^2^) (15). Following this, 2 mL of blood was withdrawn from every participant (before delivery) in an ethylenediaminetetraacetic acid and analysed for a complete blood count including haemoglobin, using an automated haematology analyser and following the manufacturer’s instructions (Sysmex KX-21, Japan).

### Sample size

The sample size was calculated considering the assumed prevalence of spontaneous PB of 13% (the ratio was 6.6:1) among all the deliveries, guided by the recent reports from Ethiopia (16). Assuming a type I error of 5% and adequate power of 80% (β = 0.2), based on the results of our previous meta-analysis (17), we assumed that 40% of the women who had a PB and 30% of women who had no PB would have anaemia, which resulted in a sample size of 1,716, considering that 10% of the women might not respond or might have incomplete data. The sample size was calculated using the OpenEpi Menu (18).

### Statistics

Data were entered into a computer, and SPSS for Windows was used for data analysis. Continuous data were checked for normality using the Shapiro–Wilk test. Descriptive statistical [mean (standard deviation), median (interquartile range), frequency, and percentage] were used to present the characteristics of the participants. The Mann-Whitney test was used to compare the median (interquartile range) between the two groups. A logistic regression analysis was performed with spontaneous PB as the dependent factor. The covariates (independent factors) were the sociodemographic, medical, and obstetric factors, age of women, parity, education, residence, occupation, antenatal care status, history of previous miscarriages/PB, gestational age, interpregnancy interval, BMI, haemoglobin level (before delivery), and sex of the infant as independent factors. Variables with a *p*-value of <0.2 were entered into the multivariable logistic regression model using the backward stepwise method (likelihood ratio). The crude odds ratio (COR), adjusted odds ratio (AOR), and 95% CI were computed to show the strength of the association. A two-sided *p*-value of < 0.05 was considered statistically significant.

## Results

A total of 1,716 parturient women were enrolled in the study. The median (interquartile range) of their age, parity, IPI, BMI, and haemoglobin was 27 (23–32) years, 2 (1–4), 23 (15–23) months, 26.1 (23.3–27.5) kg/m^2^, and 10.5 (9.8–11.4) g/dl, respectively. Of the 1,716 women, 939 (54.7%) were educated up to at least secondary level (8 years), 1,584 (92.3%) attended at least two antenatal visits, and 255 (14.9%) were obese. The details of the sociodemographic characteristics are shown in Table 1.

**TABLE 1:** Frequency and proportion of pregnant women in Khartoum, Sudan, 2020.

Variables	Frequency (*n* = 1,716)	Proportion (100)
**Education level**		
≥ Secondary	939	54.7
< Secondary	777	45.3
Antenatal care		
≥ Two visits	1,584	92.3
<Two visits	132	7.7
**Occupation**		
Housewife	1,509	87.9
Employed	207	12.1
**History of miscarriage/preterm birth**		
Yes	389	22.7
No	1,327	77.3
**Body mass index**		
Underweight	21	1.2
Normal weight	759	44.2
Overweight	681	39.7
Obese	255	14.9
**Anaemia**		
Yes	1,128	65.7
No	5,888	34.3
**Gender**		
Men	860	50.1
Women	856	49.9

Of the 1,716 parturient women, 21 (1.2%) were underweight, 759 (44.2%) were of normal weight, 681 (39.7%) were overweight, and 255 (14.9%) were obese.

Approximately two-thirds (65.7%) of the women had anaemia and six (0.3%) had severe anaemia. Parturient women were classified into seven groups according to haemoglobin levels, as follows: < 8, 8–8.9, 9–9.9, 10–10.9, 11–11.9, 12–13, and > 13 g/dl. Their numbers and proportions are shown in Table 2.

**TABLE 2:** Comparing sociodemographic and clinical variables between mothers with preterm birth and mothers with term birth in Khartoum, Sudan, 2020.

Variables	Preterm birth (283)	Term birth (1,433)	OR (95%CI)	*P*
Age, years	29.0 (24.0–34.0)	27.0 (22.0–32.0)	1.04 (1.02–1.06)	< 0.001
Parity	3.0 (1.0–5.0)	2.0 (1.0–4.0)	1.13 (1.08–1.19)	< 0.001
Interpregnancy interval, months	21.0 (15.0–37.0)	23.0 (15.0–36.0)	1.0 (0.99–1.01)	0.459
**Miscarriage/preterm birth**				
Yes	82 (29.0)	307 (21.4)	1.49 (1.12–1.99)	0.007
No	201 (71.0)	1,126 (78.6)	Reference	
**Occupation**				
Housewife	249 (88.0)	1,260 (87.9)	Reference	
Employee	34 (12.0)	173 (12.1)	1.01 (0.67–1.48)	1.000
**Antenatal care**				
≥Two visits	295 (91.5)	1,325 (92.5)	Reference	
<Two visits	24 (8.5)	108 (7.5)	1.13 (0.71–1.80)	0.625
**Body mass index**				
Underweight	3 (1.3)	18 (1.3)	0.61 (0.43–0.88)	0.008
Normal weight	117 (41.3)	642 (44.8)	Reference	
Overweight	105 (37.1)	576 (40.2)	0.65 (0.16–1.98)	0.375
Obese	58 (20.5)	197 (13.7)	0.61 (0.43–0.88)	0.009
**Maternal diseases**				
Yes	36 (12.7)	111 (7.7)	1.73 (1.16–2.58)	0.010
No	247 (87.3)	1,322 (92.3)	Reference	
**Gender**				
Women	717 (50.0)	139 (49.1)	Reference	
Men	716 (50.0)	144 (50.9)	0.96 (0.74–1.24)	0.778
**Hemoglobin level, g/dl**				
< 8.0	1 (0.4)	24 (2.7)	0.16 (0.22–1.23)	0.079
8.0–8.99	14 (4.9)	123 (8.6)	0.44 (0.24–0.83)	0.011
9.0–9.99	44 (15.5)	278 (19.4)	0.62 (0.40–0.95)	0.029
10.0–10.99	81 (28.6)	563 (39.3)	Reference	
11.0–11.99	61 (21.6)	240 (16.7)	0.56 (0.39–0.82)	0.002
12.0–13.0	60 (21.2)	147 (10.3)	1.60 (1.1–2.42)	0.024
> 13.0	22 (7.8)	58 (4.0)	1.49 (0.84–2.62)	0.165

Of the 1,716 parturient women, 283 (16.5%, 95% CI = 14.7–18.2) had a PB. Compared to women who had a term birth, women who had a PB had significantly higher age, higher parity, more history of miscarriage/PB, and were more likely to be obese (see Table 2). The median (IQR) of the haemoglobin level was significantly (Mann-Whitney test) higher in women with PTB [11.3 (10–12) g/dl vs. 10.3 (9.7–11.2), *P* < 0.001]. Moreover, compared to women who had a term birth, women who had a PB are less likely to be anaemic [140/283 (49.5%) vs. 988/1,433 (68.9%), *P* < 0.001]. In multivariable logistic regression, parity (AOR = 1.15, 95% CI = 1.09–1.21, *P* < 0.001) was positively associated with PB. Anaemia was associated with reduced OR of PB (AOR = 0.4, 95% CI = 0.32–0.54, as seen in Table 3).

**TABLE 3:** Logistic regressions of sociodemographic and clinical variables associated with preterm birth in Khartoum, Sudan, 2020.

Variables	Adjusted	
	
	AOR (95% CI)	*P*
Age, years	0.99 (0.971.03)	0.958
Parity	1.15 (1.09–1.21)	<0.001
Hemoglobin level, g/dl*	1.39 (1.26–1.53)	<0.001
**Miscarriage/preterm birth**		
Yes	1.33 (0.99–1.80)	0.056
No	Reference	
**Maternal disease**		
Yes	0.66 (0.44–1.01)	0.056
No		
**Body mass index**		
Underweight	0.85 (0.239–3.073)	0.813
Normal weight	Reference	
Overweight	0.880 (0.649–1.193)	0.409
Obese	1.133 (0.753–1.706)	0.548
Hemoglobin level, g/dl*		
< 8.0	0.13 (0.01–1.03)	0.053
8.0–8.99	0.41 (0.22–0.77)	0.006
9.0–9.99	0.59 (0.38–0.91	0.019
10.0–10.99	Reference	
11.0–11.99	0.53 (0.36–0.77)	0.001
12.0–13.0	1.62 (1.06–2.45)	0.023
>13.0	1.48 (0.83–2.63)	0.175
Anaemia*		
Yes	0.41 (0.32–0.54)	<0.001
No	Reference	

*Were entered one by one in the model, AOR, adjusted odds ratio; CI, confidence interval.

**FIGURE 1:**
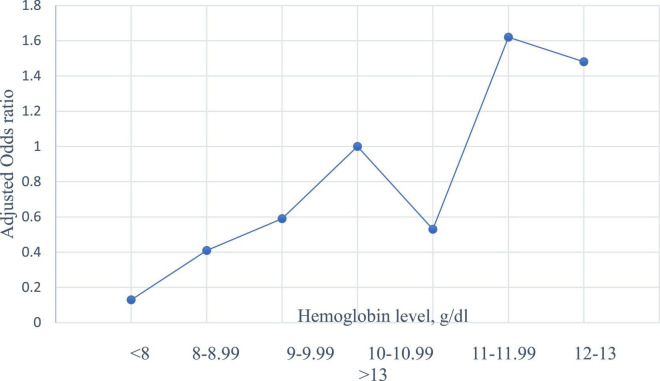
The adjusted odds ratio of haemoglobin level and preterm birth in Khartoum, Sudan 2020.

Compared to those with haemoglobin levels of 10–10.9 g/dl, parturient women with haemoglobin levels of 8–8.9 (AOR = 0.41, 95% CI = 0.22–0.77, *P* = 0.006), 9–9.9 (AOR = 0.59, 95% CI = 0.38–0.91, *P* = 0.019), and 11–11.9 g/dl (AOR = 0.53, 95% CI = 0.36–0.77, *P* = 0.002) were at lower risk of PB. Parturient women with haemoglobin levels of 12–13 g/dl were at a higher risk of PB (AOR = 1.62, 95% CI = 1.06–2.45, *P* = 0.23). There was no significant association between women with low haemoglobin levels < 8 g/dl and women with haemoglobin levels > 13.g/dl and PB (see Table 3 and Figure 1).

## Discussion

The prevalence (16.5%) of spontaneous PB in our study was found to be comparable to the prevalence of PB in other African countries, e.g., 16.9% in Ethiopia (16), 18.3% in Kenya (7), and 16.8% in Nigeria (19). However, a prevalence of 16.5% was outside the range (9.5–15.8%) reported by WHO for sub-Saharan Africa (20). The differences in the prevalence of PB could be explained by the differences in the methods (definition, inclusion criteria, and exclusion criteria) and risk factors as well as differences in social and other factors. Moreover, it was a hospital-based study that might not reflect the nature of the overall community. This might offer a plausible explanation for the high prevalence of PB in this study, especially since there is a high rate of home deliveries in Sudan (21).

In the current study, age, history of miscarriage/PB, education, ANC, BMI, duration, and IPI were not associated with PB. Similar findings were reported in other studies, which showed that IPI was not associated with PB (7). Our findings contrast with several studies showing that rural residence, short interpregnancy interval, presence of chronic illness (6), maternal age (8, 19), education, failure to attend antenatal care clinic, previous abortion (22), and previous PB were associated with PB (7).

Our results showed that, compared to those with haemoglobin levels (at the time of the delivery) of 10–10.99 g/dl, women with haemoglobin levels of 8–8.99 (AOR = 0.414), 9–9.99 (AOR = 0.59), and 11–11.99 (AOR = 0.53) g/dl were at lower risk of PB. Women with haemoglobin levels of 12–13 g/dl were at a higher risk of PB (AOR = 1.62). Previous studies refuted any association between high haemoglobin levels and PB (23, 24). In China, a high haemoglobin level in the second trimester has been shown to carry an increased risk of PB (13). Zhou et al. reported a slightly increased risk of PB associated with high haemoglobin levels (14). In Turkey, incidences of PB were significantly higher for both high and low haemoglobin levels (25). Moreover, it has been found that both low and high haemoglobin concentrations tend to be associated with an increased risk of PB, in a U-shaped pattern (26). Similarly, previous studies reported that women with high haemoglobin levels had higher risks of PB (27, 28). Interestingly, previous studies reported a U-shaped curve for the risk of PB against maternal haemoglobin concentrations (29, 30). During pregnancy, haemoglobin levels decrease due to an increase (expansion) in plasma volume. This results in a reduction in blood viscosity, which can interfere with the proper placental perfusion (31). Thus, a high haemoglobin level during pregnancy could lead to placental infarcts, poor functionality, and PB.

In Kenya (7), anaemia was not found to be associated with PB. We previously showed that the risk of PB increases significantly with anaemia (especially the severe form) (32). In a recent (2020) meta-analysis of 58 studies including a total of 134,801 women, anaemic women were found to be at higher risk of PB (8). A previous (2019) meta-analysis (of 117 studies including a total of 4,127,430 pregnancies), revealed that maternal anaemia was significantly associated with PB (12). Several mechanisms may explain the increased risk of PB in anaemia. Hypoxia and an increase in the level of norepinephrine concentrations can lead to maternal and foetal stress. This may stimulate the secretion of the corticotrophin-releasing hormone. Moreover, anaemia may also increase the risk of maternal infection, which is a known predisposing factor for PB (33). Amino acids, which can be influenced by maternal protein and quality of protein intake, play a significant role in the placenta’s function, foetus growth, and development. Low maternal protein intake can be important in reducing haemoglobin synthesis and in causing placental insufficiency. High protein intake may cause an excess of haemoglobin synthesis, leading to a relative excess of haemoglobin concentration. Moreover, plasma ammonia toxicity is potentially responsible for intrauterine growth restriction and excessive production of the metabolites of amino acids, hindering the development of the foetus (34). Also, there is accumulating evidence for changes in the maternal microbiota that might be associated with PB (35).

The study had some limitations: While there may be differential recall bias in women, haemoglobin in the first and second trimesters was not checked, serum ferritin and inflammatory biomarkers were not investigated, and several other factors and haemoglobinopathies/thalassaemia were not assessed in our cohort. These factors [e.g., malaria (6), HIV (19, 22)] and alcohol consumption (16) have been reported to be associated with PB. The prevalence of HIV and malaria is low in Khartoum. Smoking and alcohol consumption are not common in women in Sudan. Due to the sensitivity of such topics, cooperation from women may have been reduced or lost if they were asked about smoking or consuming alcohol.

## Conclusion

Haemoglobin levels have different effects on the risk of PB; while some haemoglobin levels were associated with a lower risk of PB; other haemoglobin levels were associated with an increased risk or were not associated with PB. A large longitudinal study assessing other factors (especially inflammatory factors) is required.

## Data availability statement

The original contributions presented in this study are included in the article/supplementary material, further inquiries can be directed to the corresponding author.

## Ethics statement

The studies involving human participants were reviewed and approved by the Ethics Committee at the Department of Obstetrics and Gynaecology, Faculty of Medicine, University of Khartoum, Sudan (reference number: 2019/09). All procedures performed in the study were in accordance with the ethical standards of the institutional and/or National Research Committee and with the 1964 Helsinki declaration and its later amendments. The patients/participants provided their written informed consent to participate in this study.

## Author contributions

AE and NA: conceptualisation and writing—original draft, review, and editing. AE and DR: data curation. IA: formal analysis. AE: investigation. IA and OA-W: methodology. DR: project administration. All authors have read and agreed upon the published version of the manuscript.

## Conflict of interest

The authors declare that the research was conducted in the absence of any commercial or financial relationships that could be construed as a potential conflict of interest.

## Publisher’s note

All claims expressed in this article are solely those of the authors and do not necessarily represent those of their affiliated organizations, or those of the publisher, the editors and the reviewers. Any product that may be evaluated in this article, or claim that may be made by its manufacturer, is not guaranteed or endorsed by the publisher.

## References

[1] Born too Soon. Linked to “Born too Soon: The Global Action Report on Preterm Birth.” Country Data and Rankings for Preterm Birth EMBARGO Until May 2nd 2012. Geneva: World Health Organization (2012).

[2] Chawanpaiboon S Vogel JP Moller AB Lumbiganon P Petzold M Hogan D Global, regional, and national estimates of levels of preterm birth in 2014: a systematic review and modelling analysis. *Lancet Glob Heal.* (2019) 7:e37–46. 10.1016/S2214-109X(18)30451-0 PMC6293055 30389451

[3] Mondal MNI Hossain MK Ali MK. Factors influencing infant and child mortality: a case study of Rajshahi district, Bangladesh. *J Hum Ecol.* (2009) 26:31–9.

[4] Goldenberg RL Culhane JF Iams JD Romero R. Epidemiology and causes of preterm birth. *Lancet.* (2008) 371:75–84. 10.1016/S0140-6736(08)60074-4 18177778 PMC7134569

[5] Zainal H Dahlui M Soelar SA Su TT. Cost of preterm birth during initial hospitalization: a care provider’s perspective. *PLoS One.* (2019) 14:e0211997. 10.1371/journal.pone.0211997 31237874 PMC6592503

[6] Aregawi G Assefa N Mesfin F Tekulu F Adhena T Mulugeta M Preterm births and associated factors among mothers who gave birth in Axum and Adwa Town public hospitals, Northern Ethiopia, 2018. *BMC Res Notes.* (2019) 12:640. 10.1186/s13104-019-4650-0 31578146 PMC6775657

[7] Wagura P Wasunna A Laving A Wamalwa D Ng’ang’a P. Prevalence and factors associated with preterm birth at kenyatta national hospital. *BMC Pregnancy Childbirth.* (2018) 18:107. 10.1186/s12884-018-1740-2 29673331 PMC5909235

[8] Laelago T Yohannes T Tsige G. Determinants of preterm birth among mothers who gave birth in East Africa: systematic review and meta-analysis. *Ital J Pediatr.* (2020) 46:10. 10.1186/s13052-020-0772-1 31992346 PMC6988288

[9] Aseidu EK Bandoh DA Ameme DK Nortey P Akweongo P Sackey SO Obstetric determinants of preterm delivery in a regional hospital. Accra, Ghana 2016. *BMC Pregnancy Childbirth.* (2019) 19:248. 10.1186/s12884-019-2404-6 31307408 PMC6631734

[10] Abadiga M Mosisa G Tsegaye R Oluma A Abdisa E Bekele T. Determinants of adverse birth outcomes among women delivered in public hospitals of Ethiopia, 2020. *Arch Public Health.* (2022) 80:12. 10.1186/S13690-021-00776-0 34983656 PMC8728986

[11] Abadiga M Wakuma B Oluma A Fekadu G Hiko N Mosisa G. Determinants of preterm birth among women delivered in public hospitals of Western Ethiopia, 2020: unmatched case-control study. *PLoS One.* (2021) 16:e0245825. 10.1371/JOURNAL.PONE.0245825 33493193 PMC7833256

[12] Jung J Rahman MM Rahman MS Swe KT Islam MR Rahman MO Effects of hemoglobin levels during pregnancy on adverse maternal and infant outcomes: a systematic review and meta-analysis. *Ann N Y Acad Sci.* (2019) 1450:69–82. 10.1111/nyas.14112 31148191

[13] Zhang Y Li Z Li H Jin L Zhang Y Zhang L Maternal haemoglobin concentration and risk of preterm birth in a Chinese population. *J Obstet Gynaecol.* (2018) 38:32–7. 10.1080/01443615.2017.1325454 28741390

[14] Zhou LM Yang WW Hua JZ Deng CQ Tao X Stoltzfus RJ. Relation of hemoglobin measured at different times in pregnancy to preterm birth and low birth weight in Shanghai, China. *Am J Epidemiol.* (1998) 148:998–1006. 10.1093/oxfordjournals.aje.a009577 9829872

[15] Ota E Haruna M Suzuki M Anh DD Tho H Tam NT Maternal body mass index and gestational weight gain and their association with perinatal outcomes in Viet Nam. *Bull World Heal Organ.* (2011) 89:127–36. 10.2471/BLT.10.077982 21346924 PMC3040376

[16] Kelkay B Omer A Teferi Y Moges Y. Factors associated with singleton preterm birth in shire suhul general hospital. northern ethiopia, 2018. *J Pregnancy.* (2019) 2019:4629101. 10.1155/2019/4629101 31205788 PMC6530231

[17] Adam I Ibrahim Y Elhardello O. Prevalence, types and determinants of anemia among pregnant women in Sudan: a systematic review and meta-analysis. *BMC Hematol.* (2018) 18:31. 10.1186/s12878-018-0124-1 30455961 PMC6225563

[18] Dean AG Sullivan KM Soe MM. *OpenEpi Menu.* Available online at: http://wwww.openepi.com/Menu/OE_Menu.htm (accessed January 1, 2022). (2022)

[19] Butali A Ezeaka C Ekhaguere O Weathers N Ladd J Fajolu I Characteristics and risk factors of preterm births in a tertiary center in Lagos, Nigeria. *Pan Afr Med J.* (2016) 24:1. 10.11604/pamj.2016.24.1.8382 27583065 PMC4992393

[20] Blencowe H Cousens S Chou D Oestergaard M Say L Moller AB Born too soon: the global epidemiology of 15 million preterm births. *Reprod Health.* (2013) 10:S2. 10.1186/1742-4755-10-S1-S2 24625129 PMC3828585

[21] Abbaker AO Salih Y Ali AA Imam AM Abdulla EA Adam I. Use of institutional delivery services in Kassala, eastern Sudan. *Int J Gynecol Obstet.* (2013) 123:79–80. 10.1016/j.ijgo.2013.04.018 23850365

[22] Mekonen DG Yismaw AE Nigussie TS Ambaw WM. Proportion of preterm birth and associated factors among mothers who gave birth in debretabor town health institutions, northwest, Ethiopia 1. *BMC Res Notes.* (2019) 12:2. 10.1186/s13104-018-4037-7 30602378 PMC6317243

[23] Ren A Wang J Ye RW Li S Liu JM Li Z. Low first-trimester hemoglobin and low birth weight, preterm birth and small for gestational age newborns. *Int J Gynecol Obstet.* (2007) 98:124–8. 10.1016/j.ijgo.2007.05.011 17585914

[24] Scanlon KS Yip R Schieve LA Cogswell ME. High and low hemoglobin levels during pregnancy: differential risks for preterm birth and small for gestational age. *Obstet Gynecol.* (2000) 96:741–8. 10.1016/S0029-7844(00)00982-0 11042311

[25] Çakmak BD Türker ÜA Öztaş S Arık M Üstünyurt E. The effect of first trimester hemoglobin levels on pregnancy outcomes. *Turk J Obstet Dern Derg.* (2018) 15:165–70. 10.4274/tjod.87269 30202626 PMC6127473

[26] Malhotra M Sharma JB Batra S Sharma S Murthy NS Arora R. Maternal and perinatal outcome in varying degrees of anemia. *Int J Gynaecol Obstet.* (2002) 79:93–100. 10.1016/s0020-7292(02)00225-4 12427391

[27] Gonzales GF Steenland K Tapia V. Maternal hemoglobin level and fetal outcome at low and high altitudes. *Am J Physiol.* (2009) 297:1105–10. 10.1152/ajpregu.00275.2009 19741055 PMC2777782

[28] Chang S O’Brien K Nathanson MS Mancini J Witter FR. Hemoglobin concentrations influence birth outcomes in pregnant African-American adolescents. *J Nutr.* (2003) 133:2348–55. 10.1093/jn/133.7.2348 12840205

[29] Dewey KG Oaks BM. U-shaped curve for risk associated with maternal hemoglobin, iron status, or iron supplementation. *Am J Clin Nutr.* (2017) 106:1694–702S. 10.3945/ajcn.117.156075 29070565 PMC5701708

[30] Zhang X Xu Q Yang Y Wang L Liu F Li Q Preconception Hb concentration and risk of preterm birth in over 2⋅7 million Chinese women aged 20-49 years: a population-based cohort study. *Br J Nutr.* (2018) 120:508–16. 10.1017/S0007114518001721 29986785

[31] Mani S Duffy TP. Anemia of pregnancy. *Clin Perinatol.* (1995) 22:593–607. 8521683

[32] Ali AA Rayis DA Abdallah TM Elbashir MI Adam I. Severe anaemia is associated with a higher risk for preeclampsia and poor perinatal outcomes in Kassala hospital, eastern Sudan. *BMC Res Notes.* (2011) 4:311. 10.1186/1756-0500-4-311 21867566 PMC3224576

[33] Allen L. Biological mechanisms that might underlie iron’s effects on fetal growth and preterm birth. *J Nutr.* (2001) 131:604–15S. 10.1093/JN 11160591

[34] Herring CM Bazer FW Johnson GA Wu G. Impacts of maternal dietary protein intake on fetal survival, growth, and development. *Exp Biol Med.* (2018) 243:525. 10.1177/1535370218758275 29466875 PMC5882021

[35] Nuriel-Ohayon M Neuman H Koren O. Microbial changes during pregnancy, birth, and infancy. *Front Microbiol.* (2016) 7:1031. 10.3389/FMICB.2016.01031 27471494 PMC4943946

